# Apical Negative Pressure irrigation presents tissue compatibility in immature teeth

**DOI:** 10.1590/1678-7757-2016-0599

**Published:** 2017

**Authors:** Carolina Maschietto Pucinelli, Léa Assed Bezerra da Silva, Nestor Cohenca, Priscilla Coutinho Romualdo, Raquel Assed Bezerra da Silva, Alberto Consolaro, Alexandra Mussolino de Queiroz, Paulo Nelson

**Affiliations:** 1 Universidade de São Paulo, Faculdade de Odontologia de Ribeirão Preto, Departamento de Clínica Infantil, Ribeirão Preto, SP, Brasil.; 2 University of Washington & Seattle Children's, Department of Endodontics, Seattle, USA.; 3 Universidade de São Paulo, Faculdade de Odontologia de Bauru, Departamento de Cirurgia, Estomatologia, Patologia e Radiologia, Bauru, SP, Brasil.

**Keywords:** Apical negative pressure irrigation, Apical periodontitis, Apical positive pressure irrigation, Immature teeth

## Abstract

**Aim:**

To compare the apical negative pressure irrigation (ANP) with conventional irrigation in the teeth of immature dogs with apical periodontitis.

**Methods:**

Fifty-two immature pre-molar root canals were randomly assigned into 4 groups: ANP (n=15); conventional irrigation (n=17); healthy teeth (control) (n = 10); and teeth with untreated apical periodontitis (control) (n=10). After induction of apical periodontitis, teeth were instrumented using EndoVac^®^ (apical negative pressure irrigation) or conventional irrigation. The animals were euthanized after 90 days. The sections were stained by HE and analyzed under conventional and fluorescence microscopy. TRAP histoenzymology was also performed. Statistical analyses were performed with the significance level set at 5%.

**Results:**

There was difference in the histopathological parameters between ANP and conventional groups (p<0.05). The ANP group showed a predominance of low magnitude inflammatory infiltrate, a smaller periodontal ligament, and lower mineralized tissue resorption. There were no differences in the periapical lesion extensions between the ANP and conventional groups (p>0.05). However, a lower number of osteoclasts was observed in the ANP group (p<0.05).

**Conclusion:**

The EndoVac^®^ irrigation system presented better biological results and more advanced repair process in immature teeth with apical periodontitis than the conventional irrigation system, confirming the hypothesis.

## Introduction

Infection control in endodontic therapy is extremely important to achieve a successful outcome in teeth with apical periodontitis^7^ ^,^ ^16^ ^,^ ^25^ ^,^ ^27^. This can be obtained through the steps involved in the endodontic treatment^15^, including irrigation^8^. The association between irrigation and biomechanical preparation optimizes the cleaning of root canals^6^ ^,^ ^22^.

Although the conventional irrigation system is widely used^12^, in 2007 a novel system called EndoVac^®^ (Discus Dental, Culver City, CA, USA) was launched to the dental market. Instead of positive pressure, the EndoVac^®^ system uses an apical negative pressure irrigation (ANP) and has been considered a promising disinfection protocol in the endodontic literature^8^ ^,^ ^23^. Previous studies demonstrated that the ANP decreases the risk of irrigant solution extrusion through the apical foramen^9^ ^,^ ^14^ ^,^ ^26^. This system enables the circulation of irrigant solution to all working lengths (WL)^2^ and facilitates microbiological control^13^ ^,^ ^23^. The ANP is also efficient in removing the “smear layer"^10^ ^,^ ^18^ and debris, mostly at the apical third of a root canal^1^ ^,^ ^4^ ^,^ ^10^ ^,^ ^11^.

The anatomy of the apical third in the root canals of immature teeth increases the risk of accidental injection of irrigant solution into the periapical tissues^3^ ^,^ ^26^. However, an *in vitro* study demonstrated that open apex teeth had similar extrusion to closed apex teeth when the ANP was used^20^.

To date, no *in vivo* studies comparing the EndoVac^®^ system with the conventional irrigation system separately in immature teeth have been published. Therefore, the aim of this *in vivo* study was to perform the histopathological and histoenzymological evaluation to compare the EndoVac^®^ system with the conventional irrigation in immature dog teeth with experimentally induced apical periodontitis. The hypothesis is that ANP presented better biological results in immature teeth with apical periodontitis than the conventional irrigation system.

## Material and methods

This research project was approved by the Institutional Animal Ethics Committee (#006/2012). All the experimental procedures were performed as in our previous studies^8^ ^,^ ^24^.

Three beagle dogs, 4 months old, were used. Upper (second and third) and lower (second, third, and fourth) immature premolars were included. Fifty-two roots were randomly divided into 4 groups, as follows:

ANP (Apical Negative Pressure): (n=15).

Conventional Irrigation (Positive Pressure): (n=17).

Healthy Teeth (Negative Control): (n = 10)

Teeth with untreated apical periodontitis (Positive Control): (n = 10).

All teeth had an X-ray taken to confirm an open apex. The coronal access was performed in the EndoVac^®^, conventional, and apical periodontitis groups. After pulp tissue removal, root canals were left exposed in the oral cavity for 7 days for microbial contamination, as recommended by Leonardo, et al.^17^ (1993). In order to promote the induction of apical periodontitis, the pulp chamber was sealed with zinc oxide eugenol cement (SS White, Rio de Janeiro, RJ, Brazil). Bone thinning occurs between 15 and 25 days in immature dog teeth^17^.

After this period, a rubber dam was used to isolate the teeth and the temporary restoration was removed. Root canal disinfection was performed 3 mm shorter than the radiographic apex, followed by WL determination, established 1 mm shorter than the radiographic apex. The complementation of root canal disinfection was performed at the WL. ANP and conventional groups were instrumented with K-type files to the WL. At each file exchange, root canals were irrigated with 10 mL of NaOCl 2.5% for both the EndoVac^®^ system and conventional system.

The recommended protocol for the EndoVac^®^ system includes 2 main steps: macro-irrigation and micro-irrigation. In this particular study with immature teeth, canals were irrigated using the macro-cannula only after determining the apical size of the canal due to the large apical size, as previously published^23^. Finally, root canals were irrigated with saline solution, dried with absorbent paper points and sealed with ProRoot MTA (Dentsply Tulsa Dental, Johnson City, TN, USA) and silver amalgam (Sybraloy, Kerr Corporation, Orange, CA, USA).

### HE-staining

After 90 days, the animals were euthanized. The maxillas and mandibles with teeth were dissected and sectioned to obtain individual roots. The histotechnical procedures were performed as previously published^5^ ^,^ ^8^.

The HE-stained sections were analyzed in an Axio Imager. M1 microscope (Zeiss, Göttingen, Germany), using scores based on the following histopathological parameters: (a) inflammatory infiltrate: absent or mild (score 1), moderate or severe (score 2); (b) thickness of periodontal ligament: normal (score 1), slightly increased (score 2), moderately increased (score 3), or severely increased (score 4); and (c) process of resorption of mineralized tissues: absent (score 1) or present (score 2). In addition, descriptions of the apical and periapical regions were conducted for each group.

### Fluorescence microscope morphometry

In the ANP, conventional, and apical periodontitis groups, the area of the periapical lesion was measured in square millimeters in the HE-stained sections using an Axio Imager.M1 microscope at x1.25 magnification and operating in the fluorescence mode, as previously described^5^ ^,^ ^19^ ^,^ ^24^.

In the healthy teeth group, the thickness of the healthy periodontal ligament area was measured by drawing a line perpendicular to the root apex, located 0.5 mm above the opening of each specimen, to delimit the maximum height of the measured area.

### Tartrate-resistant acid phosphatase histoenzymology (TRAP)

The TRAP activity was performed to mark the osteoclasts^8^ ^,^ ^19^. The sections were deparaffinized, hydrated, and placed in a solution of 50% ethanol/acetone for 1 minute and dried at room temperature. Next, a buffer solution containing acetic acid, dimethylformamide, Fast Red, and phosphoric acid naphthol AS-BI (Sigma-Aldrich Corporation, St. Louis, MO, USA) was pipetted over the sections, which were maintained at 37°C for 40 minutes protected from light. The counter-stain with Fast Green was performed.

The samples were examined under the Axio Imager. M1 microscope under conventional light to count the number of multinucleate TRAP-positive cells present in the resorption lacunae that were in direct contact with the alveolar bone around the periapical lesion.

There was an invagination of partially mineralized connective tissue into the root canal in the ANP specimens. In the healthy specimens, the osteoclast count region was established as described for the fluorescence microscope morphometry. All results were expressed in cell numbers.

### Statistical Analysis

The GraphPad Prism 5.a (GraphPad Software Inc., San Diego, CA, USA) was used for statistical analyses. Chi-square or Fisher's exact test were used to evaluate the scores. One-way ANOVA with Tukey's *post hoc* test was used to evaluate mean difference. The level of significance was set at 5%.

## Results

### Microscopic analysis of apical and periapical regions

**Figure 1:**
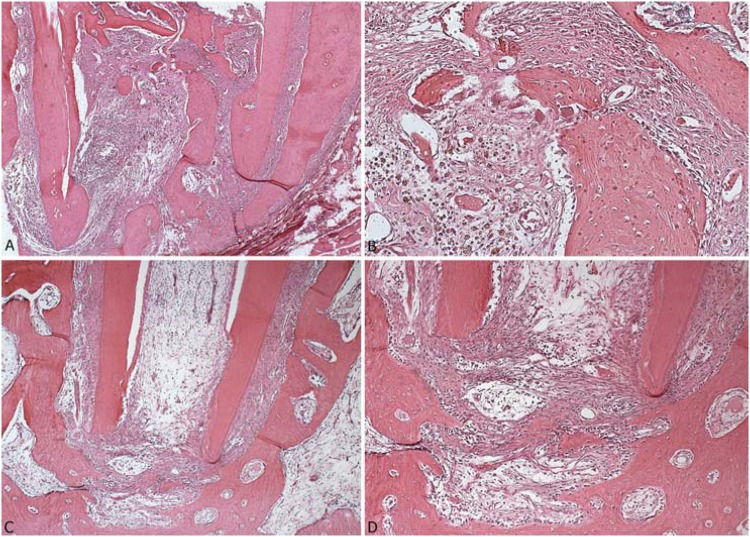
Representative photomicrographs of the ANP group, 90 days after the endodontic treatment, in conventional light microscopy: (A) Panoramic view of the periapical and apical regions showing intense invagination of the connective tissue into the root canal (HE, Zeiss, 5X). (B) Detail of panel A: part of the invaginated tissue after the mineralization (HE, Zeiss, 20X). (C) Panoramic view of the periapical and apical regions showing that the periodontal ligament was slightly increased (HE, Zeiss, 5X). (D) Photomicrography of the periapical and apical regions with fibers, vessels, and mild inflammatory cells (HE, Zeiss, 10X). HE = hematoxylin & eosin

The ANP group presented mixed and diffused inflammatory infiltrate, ranging from mild (53.3%) to moderate (26.7%). The periapical region showed rich neovascularization and better repair process with fibroblast proliferation. There was partially mineralized connective tissue in the region of the large apical foramen to the middle third of the root canal. This tissue originated from the periodontal tissue that invaginated into the root canal in 53.6% of the cases, with a dense presence of fibroblasts and blood vessels. In 80% of the specimens there was no dentin, bone, or cementum resorption (Figure 1).

**Figure 2:**
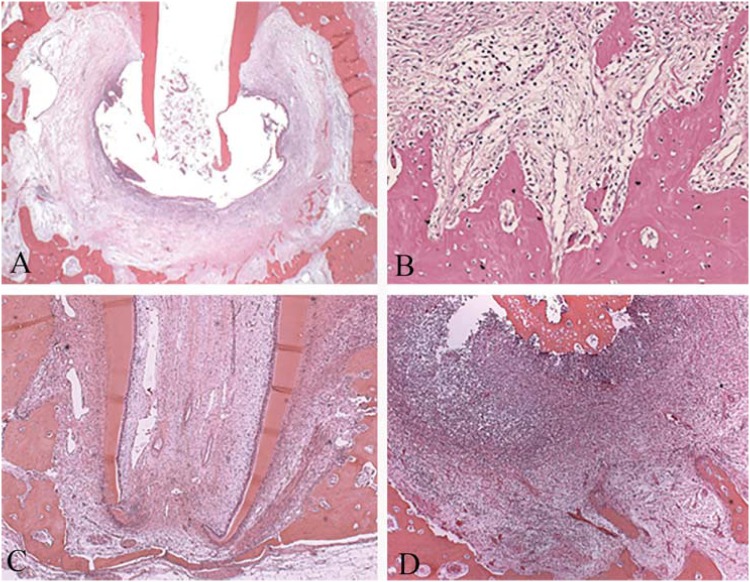
Representative photomicrographs of the conventional, healthy, and apical periodontitis groups, 90 days after the endodontic treatment, in conventional light microscopy: (A) Panoramic view of the apical and periapical regions of the conventional group showing an increased periodontal ligament (HE, Zeiss, 1.25X). (B) Detail of the alveolar bone of the conventional group with no osteoblast in its surface and presence of osteoclast (HE, Zeiss, 20X). (C) Panoramic view of the healthy teeth group with incomplete root formation, normal pulp, and an odontoblastic layer. Periodontal ligament and alveolar bone were healthy (HE, Zeiss, 10X). (D) Panoramic view of the apical periodontitis group showing a focal severe inflammatory infiltrate (HE, Zeiss, 5X). HE = hematoxylin & eosin

In the conventional group, there were mixed and diffused inflammatory infiltrate in all cases (100%). The majority of the cases (58.8%) were moderately inflamed. The periodontal ligament was severely increased in 82.4% of the cases, with areas of edema and fibrillar dissociation. In 76.5% of the cases, there was severe bone, cementum, and dentin resorption. In some cases, there were no cementoblasts on the cement surface and unrepaired root resorption was also frequent (Figure 2A and B).

All parameters (pulp tissue, odontoblast layer, periodontal ligament, and alveolar bone) in the healthy teeth group were normal (Figure 2C).

In the apical periodontitis group, the apical and periapical regions presented severely mixed and diffused inflammatory infiltrate. The periodontal ligament was severely increased with intense edema and fibrillar dissociation. In the cementum surface, the resorption areas were not repaired. The alveolar bone was distant from the root apex, indicating advanced bone resorption. There were no osteoblasts on the surface and osteoclasts were frequently present (Figure 2D).

Inflammatory infiltrate scores demonstrated significant difference between the ANP and conventional groups (p=0.03). Regarding the periodontal ligament, difference was observed (p=0.02) between the ANP and conventional groups. Statistically significant difference was also observed between these groups (p=0.003), regarding mineralized tissue resorption (Table 1).

**Table 1:** Results for Inflammatory infiltrate, thickness of periodontal ligament, and resorption of mineralized tissue between the groups

		Groups			p-Value					
Scores	EndoVac®	Conventional	Healthy	Apical	EndoVac®	EndoVac®	EndoVac®	Conventional	Conventional
				Periodontitis	x	x	x	x	x
					Conventional	Healthy	Apical	Healthy	Apical
							Periodontitis		Periodontitis
Inflammatory infiltrate									
Absent or Mild	10(66.6%)	4(23.6%)	10(100%)	0(0%)	0.03	0.06	0.001	0.0002	0.263
Moderate or Severe	5(33.4%)	13(76.4%)	0(0%)	10(100%)					
Thickness of periodontal ligament									
Normal	3(20%)	0(0%)	10(100%)	0(0%)					
Slightly increased	2(13.4%)	0(0%)	0(0%)	0(0%)					
Moderately increased	5(33.3%)	3(17.6%)	0(0%)	0(0%)	0.02	≤0.0001	0.005	≤0.0001	0.273
Severely increased	5(33.3%)	14(82.4%)	0(0%)	10(100%)					
Process of resorption of mineralized tissues									
Present	3(20%)	13(76.5%)	0(0%)	10(100%)	0.003	0.250	≤0.0001	≤0.0001	0.263
Absent	12(80%)	4(23.5%)	10(100%)	0(0%)					

### Fluorescence microscopy morphometry

**Figure 3:**
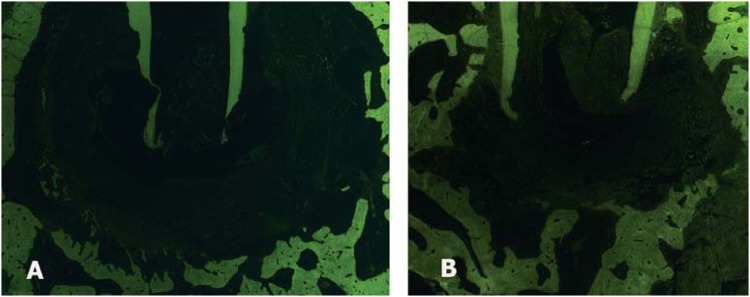
Photomicrographs of microscopic sections representing the 4 groups evaluated, 90 days after the endodontic treatment: (A) Representative photomicrographs of the conventional group, HE-Stained and observed under fluorescence microscopy (HE, Zeiss, 1.25X). (B) Representative photomicrographs of the ANP group, HE-Stained and observed under fluorescence microscopy (HE, Zeiss, 1.25X). HE = hematoxylin & eosin

The mean lesion size was 12.94 (±7.73) mm^2^ in the ANP group, 17.91 (±8.85) mm^2^ in the conventional group, and 21.47 (±1.48) mm^2^ in the apical periodontitis group. In the healthy teeth group, the periodontal ligament area in the apical region was 0.67 mm^2^ (±0.38) mm^2^. There was no significant difference between the ANP and conventional groups (p>0.05). Figure 3 shows representative photomicrographs of the different groups after fluorescence microscopy (A, B).

### TRAP histoenzymology

**Figure 4:**
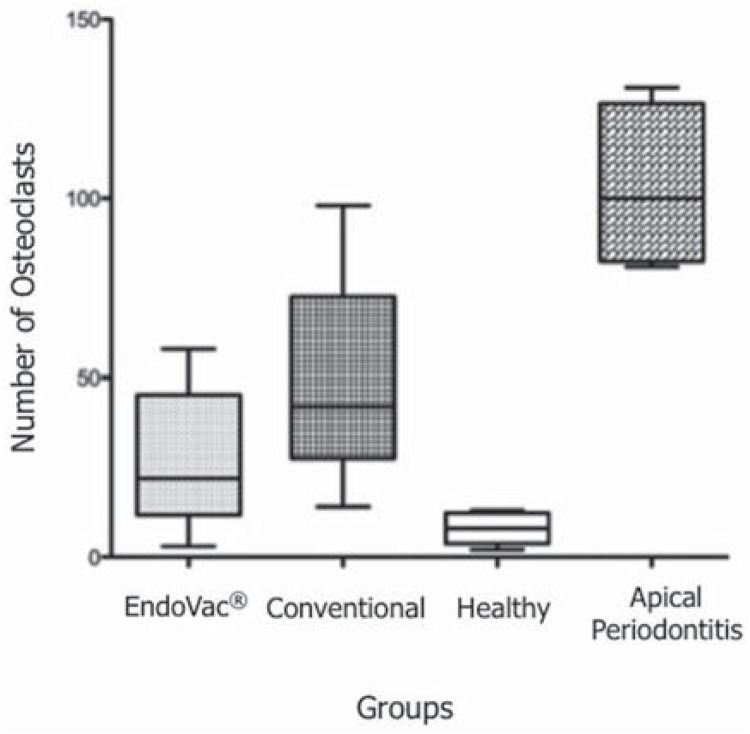
Distribution of the number of osteoclasts. The different letters represent groups with significant difference (p>0.05).

The means for the osteoclast counts were 26.25 (±18.78) for the ANP group, 50.94 (±26.74) for the conventional group, 7.9 (±4.99) for the healthy teeth group, and 103 (±23.27) for the apical periodontitis group (Figure 4). Significant difference was observed between the ANP and conventional groups (p<0.0001).

**Figure 5:**
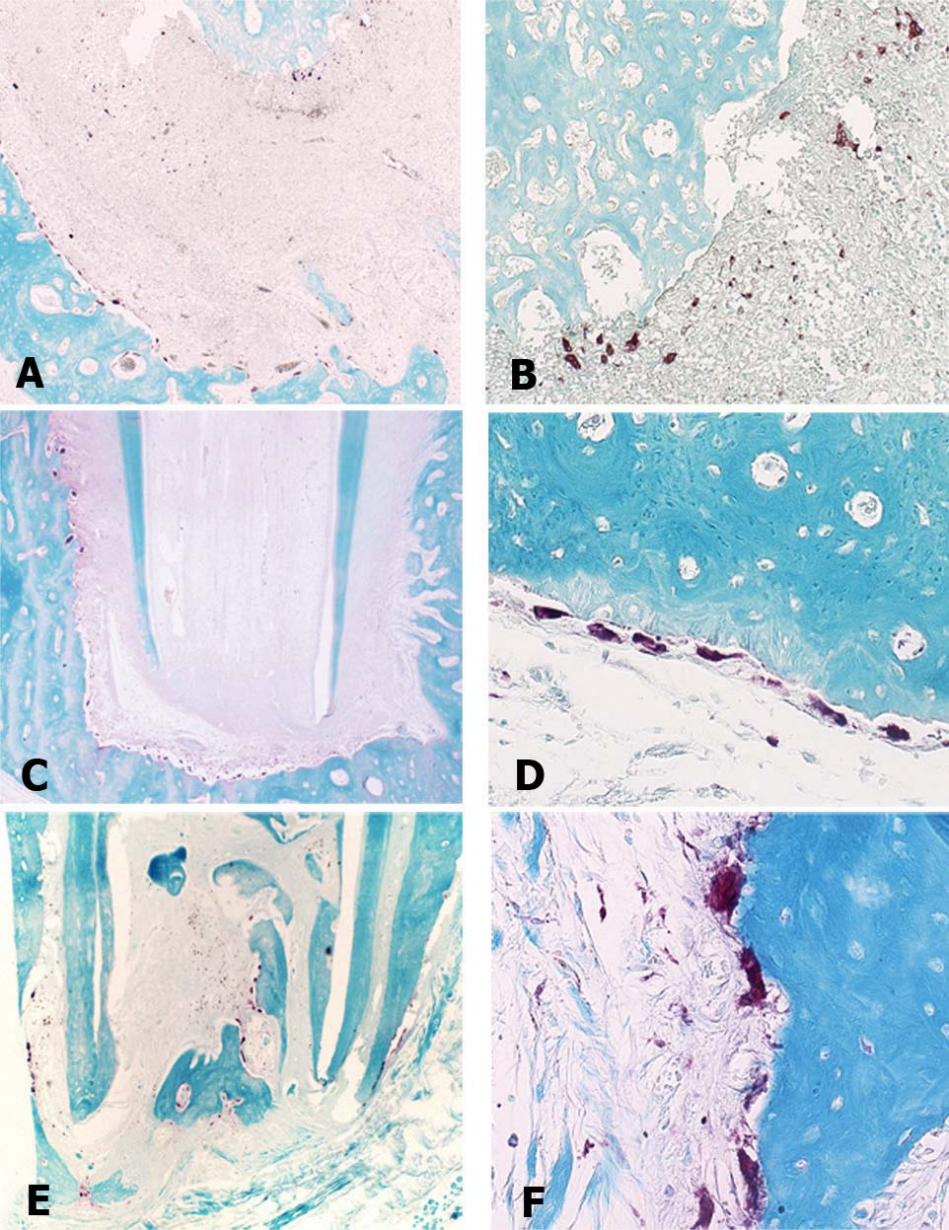
Photomicrographs of microscopic sections representing the 4 groups evaluated, 90 days after the endodontic treatment, stained by TRAP technique for identification and count of osteoclastic cells: (A) Representative photomicrographs of the Apical Periodontitis group, where it was observed that the apex and alveolar bone had intense presence of osteoclasts (Zeiss, 5X). (B) Image A detail, highlighting intense presence of osteoclasts (Zeiss, 20X). (C) Representative photomicrographs of healthy teeth group, where there is healthy tissues with reduced presence of osteoclasts (Zeiss, 5X). (D) Representative photomicrograph of the conventional irrigation group. The surface of the alveolar bone showing moderate presence of osteoclasts, albeit in larger quantity than in the ANP group (Zeiss, 40X). (E) Representative photomicrograph of the ANP group with slightly increased presence of osteoclasts (Zeiss, 5X). (F) Detail of image E, highlighting the osteoclasts in the bone surface (Zeiss, 40X).

Figure 5 shows representative photomicrographs of all groups after TRAP histoenzymology.

## Discussion

Our research group's previous study^8^ demonstrated that the ANP presented many advantages in comparison with the conventional irrigation system for teeth with closed apex. However, there are no *in vivo* studies that evaluated the EndoVac^®^ system in teeth with immature root formation, compared with conventional irrigation separately. Thus, this *in vivo* study aimed to add important information to provide scientific background for the clinical application of ANP in teeth with open apex. This is extremely important to achieve a successful outcome post-endodontic treatment in immature teeth with apical periodontitis.

This histopathological study compared two different types of root irrigation system. The initial experimental stages evaluation enables to observe an acute inflammatory response. *In vivo* methodology involves difficulties in obtaining the animals, high cost technique, as well as ethical implications. For these reasons, we decided to evaluate a longer period to observe the occurrence of persistent injury to tissues and, consequently, the inflammatory response in the late stage after use of different root irrigation systems. This methodology represents the “gold standard” of tissue response against the use of different materials or techniques. In the endodontic literature, only 2 papers^8^ ^,^ ^23^ evaluated the microscopic response of the EndoVac^®^ system *in vivo* using dog teeth. Silva, et al.^23^ (2010) compared the revascularization and the apical and periapical repair in immature dog teeth with periapical lesions after irrigation with the EndoVac^®^ system and conventional irrigation plus triantibiotic intracanal dressing. Although only the inflammatory infiltrate was significantly different between the groups, the cases treated with the ANP had a higher mineralized tissue formation in the apical region. The authors concluded that the EndoVac^®^ system might be considered a valuable disinfection protocol in immature permanent teeth with apical periodontitis, and thus intracanal antibiotics would not be necessary. The results of our study are in agreement with Silva, et al.^23^ (2010), in which EndoVac^®^ specimens presented structured connective tissue, rich vascularization, and repair process in advanced stage.

Cohenca, et al.^8^ (2015) compared the ANP irrigation with conventional irrigation and ultrasonic irrigations in dog teeth with complete root formation and apical periodontitis. The results of their microscopic analysis showed significant difference only in the inflammatory infiltrate, which was lower in the ANP specimens in comparison with conventional irrigation. The periodontal ligament thickness, resorption of mineralized tissues, size of periapical lesions, and the number of osteoclasts did not present statistically significant difference between the groups. These results do not agree with ours, probably due to the particular anatomical characteristics of teeth with incomplete root formation, which present richer vascular supply^21^ ^,^ ^29^ enabling shorter repair process.

In our study, there was a partially mineralized tissue invagination in 53.6% of the root canals 90 days after use of the ANP. The invagination of healthy periodontal tissue into the root canal in the apical region indicated that the intense microbial contamination was controlled in 53.6% of the cases. We hypothesize that the antimicrobial dressing between the appointments might increase this success rate.

There was no statistical difference between the EndoVac^®^ and conventional groups regarding the results of the post-irrigation periapical lesion area measurement in the fluorescence assessment. On the other hand, through the histopathological analysis of sections stained with HE, advanced stage tissue repair in the ANP specimens was evident. The periapical lesions that were present in some specimens of the ANP group did not completely repair and presented an extensive lesion area. This may explain the lack of significant difference between the lesion areas in the ANP and conventional groups in the assessment under fluorescence.

Bone is a dynamic tissue with continuous remodeling process. In pathological conditions, such as chronic apical periodontitis, bone resorption is greater than bone formation. One of the characteristics of this condition is the accumulation of osteoclasts in the bone resorption areas^28^. In this study we observed significant difference in the number of osteoclasts between the two types of irrigation. The average number of TRAP-positive cells was lower in EndoVac^®_^ treated teeth than in conventional irrigation. This result does not agree with that previously published by Cohenca, et al.^8^ (2015), who observed no statistical difference in the numbers of osteoclasts. This was probably due to the fact that the authors used teeth with complete root formation and closed apex as experimental model.

## Conclusion

The results from this *in vivo* study allowed us to conclude that the negative pressure irrigation (EndoVac^®^) demonstrated better biological results than the conventional irrigation in immature teeth with apical periodontitis and presented a more advanced repair process, thus confirming the hypothesis. Clinical studies should be performed in order to provide additional information for dental practice.
